# Ouabain-Induced Apoptosis in Cochlear Hair Cells and Spiral Ganglion Neurons *In Vitro*

**DOI:** 10.1155/2013/628064

**Published:** 2013-10-22

**Authors:** Yong Fu, Dalian Ding, Lei Wei, Haiyan Jiang, Richard Salvi

**Affiliations:** ^1^Department of Otorhinolaryngology, The First Affiliated Hospital, College of Medicine, Zhejiang University, Hangzhou, Zhejiang 310003, China; ^2^Center for Hearing and Deafness, University at Buffalo, Buffalo, NY 14214, USA

## Abstract

Ouabain is a common tool to explore the pathophysiological changes in adult mammalian cochlea *in vivo*. In prior studies, locally administering ouabain via round window membrane demonstrated that the ototoxic effects of ouabain *in vivo* varied among mammalian species. Little is known about the ototoxic effects *in vitro*. Thus, we prepared cochlear organotypic cultures from postnatal day-3 rats and treated these cultures with ouabain at 50, 500, and 1000 **μ**M for different time to elucidate the ototoxic effects of ouabain *in vitro* and to provide insights that could explain the comparative ototoxic effects of ouabain *in vivo*. Degeneration of cochlear hair cells and spiral ganglion neurons was evaluated by hair-cell staining and neurofilament labeling, respectively. Annexin V staining was used to detect apoptotic cells. A quantitative RT-PCR apoptosis-focused gene array determined changes in apoptosis-related genes. The results showed that ouabain-induced damage *in vitro* was dose and time dependent. 500 **μ**M ouabain and 1000 **μ**M ouabain were destructively traumatic to both spiral ganglion neurons and cochlear hair cells in an apoptotic signal-dependent pathway. The major apoptotic pathways in ouabain-induced spiral ganglion neuron apoptosis culminated in the stimulation of the p53 pathway and triggering of apoptosis by a network of proapoptotic signaling pathways.

## 1. Introduction

The sodium- and potassium-dependent adenosine triphosphatase (Na^+^/K^+^-ATPase) is an integral membrane protein and enzyme responsible for establishing or maintaining the electrochemical gradients of Na^+^ and K^+^ ions across the plasma membrane. The Na^+^/K^+^-ATPase consists of large catalytic subunits, and two major subunits are transporter *α* and transporter *β*. The transport *α* subunit plays a role as transporter, while the transport *β* subunit stabilizes the complex [1]. In mammals, three isoforms of *α* subunit (*α*1, *α*2, and *α*3) and two isoforms of *β* subunit (*β*1 and *β*2) that are needed for multiple cellular functions have been characterized. These functions include resting potential maintenance, transport enabling, cellular volume regulation, transducer signaling, and energy expenditure [2, 3].

The Na^+^/K^+^-ATPase is highly expressed in various regions of the inner ear, including cochlear hair cells (HCs), spiral ganglion neurons (SGNs), auditory nerve endings, vestibular HCs and dark cells, endolymphatic sac, epithelium on the spiral limbus, type II fibrocytes in the spiral ligament, and the marginal cells of the stria vascularis, which is a controller crucial to regulating the endolymphatic potential in the scala media [4–13]. The differential expression of the Na^+^/K^+^-ATPase presumably reflects different K^+^ and Na^+^ transport capabilities among the cell types found in the inner ear, which serves to generate the unique ionic and electrical environment of the mammalian inner ear. Studies of Na^+^/K^+^-ATPase expression in the developing inner ear have included a study of the cochlea of the gerbil, mouse, and rat, which provided detailed information about the Na^+^/K^+^-ATPase in various cell types in the inner ear in developmental stages [12–18].

Ouabain is a cardiac glycoside that can selectively inhibit the activity of Na^+^/K^+^-ATPase. Ototoxic effects of ouabain have been studied as a common model *in vivo* for studies in cochlear potentials, homeostasis, and cell death pathways in various mammalian species [19–32]. Recent studies have demonstrated that ouabain not only inhibited the sodium/potassium pump but also induced complex signaling cascades that lead to the cell apoptosis [27–33].

Previous studies also showed that local application of ouabain through the round window could selectively destroy type I SGNs while leaving HCs intact in the mouse and gerbil [27–33]. By contrast, the sensory HCs and limbal fibrocytes were damaged first when ouabain was applied via the round window or vestibular system in the guinea pig [34]. However, our recent study revealed that local application of ouabain in the adult rat destroyed only the SGNs and auditory nerve fibers at low doses, exactly as it usually does in the gerbil, whereas in our study ouabain damaged both SGNs and HCs at high concentrations [34]. Based on these observations, ototoxic effects of ouabain *in vivo *are different in a wide variety of mammalian species. We speculated that the important reason accounting for these differences was that ouabain-induced damage was both dose and time dependent, even though the degenerative mechanisms remained unclear.

In order to delineate these different ototoxic effects of ouabain *in vivo*, carefully designed *in vitro *experiments are required. However, little research has been conducted with respect to the toxicity of ouabain to the cochlea *in vitro*. Thus, the current study was designed to determine the ototoxic effects and the apoptotic pathways of ouabain in a cochlear organotypic culture system in the rat.

## 2. Materials and Methods

### 2.1. Animals

Sprague-Dawley rat pups were obtained from C harles River Laboratories (Wilmington, MA, USA). A total of 126 cochlear explants were used for this study: 60 for SGN and HC counts, 6 for exploring apoptotic signals in SGNs and HCs, and 60 for apoptosis-focused gene arrays. All experimental procedures, including housing, breeding, and use in experimental protocols, were approved by the Institutional Animal Care and Use Committee (IACUC) of the University at Buffalo, NY, USA, and conformed to the guidelines issued by the National Institutes of Health, MD, USA.

### 2.2. Cochlear Organotypic Cultures

The procedures for preparing cochlear organotypic cultures have been described in detail in our earlier publications [35–42]. In brief, to establish a collagen-gel matrix, a drop (15 *μ*L) of cool rat tail collagen (Type 1, Collaborative Biomedical Products no. 40236), together with a mixture of 3.76 mg/mL in 0.02 N acetic acid, 10× basal medium eagle (BME, Sigma B9638), and 2% sodium carbonate at a 9 : 1 : 1 ratio was placed at the center of a 35 mm^2^ diameter culture dish (Falcon 1008, Becton Dickinson) and allowed to form a gel at room temperature. Then 1.2 mL of serum-free medium, consisting of 2 g bovine serum albumin (BSA, Sigma A-4919), 2 mL Serum-Free Supplement (Sigma I-1884), 4.8 mL of 20% glucose (Sigma G-2020), 0.4 mL penicillin G (Sigma P-3414), 2 mL of 200 mM L-glutamine (Sigma G-6392), and 190.8 mL of 1× BME (Sigma B-1522) were added to the dish. Sprague-Dawley rat pups at postnatal day 3 were decapitated, and the cochleae were carefully removed and placed into Hank's Balanced Salts Solution (1X Gibco, 14175, Invitrogen, Carlsbad, CA, USA). The cochlear lateral wall and auditory nerve bundle in the center of the modiolus were dissected away, respectively, and the whole basilar membrane containing the organ of Corti and SGNs was transferred onto a collagen-gel matrix as a flat surface preparation. The cochlear explants were placed into an incubator (Forma Scientific, no. 3029) and maintained at 37°C in 5% CO_2_ overnight for recovery. In the next day, the serum-free medium was exchanged with a new medium, with or without ouabain, and 60 cochleae were randomly divided into 12 groups (*n* = 5 per group). A total of 3 groups were used as normal controls, which were cultured in standard serum-free medium without ouabain, ending at 24 h, 48 h, and 72 h, respectively. The remaining 9 groups were treated with various concentrations (50, 500, or 1000 *μ*M) of ouabain in serum-free medium for 24 h, 48 h, or 72 h, respectively.

### 2.3. Histology

At the end of the experiment, the cochlear explants were fixed with 10% formalin in PBS for 2 h. After fixation, specimens were rinsed in 0.01 M PBS and then incubated with a monoclonal mouse antibody against neurofilament 200 in 1% Triton X 100 and 5% goat serum in PBS (1 : 200) for 48 h. Cochlear tissue was rinsed three times in PBS for 15 min with agitation and incubated with 1% Triton X 100 and 5% goat serum in PBS plus a secondary antibody labeled with Alexa Fluor 555 (1 : 400) (goat anti-mouse IgG, Invitrogen, A21426) for 1 h at room temperature to label SGNs and their auditory nerve fibers. After rinsing with 0.1 M PBS, specimens were immersed in Alexa 488-labeled phalloidin (1 : 200) (Sigma P1951) in PBS for 1 h to label the stereocilia and cuticular plate of the HCs. Cochlear tissue was washed three times in PBS and mounted on glass slides in glycerin.

To explore the apoptotic signals in HCs and SGNs, six additional cochlear explants were cultured with ouabain at a concentration of 50 *μ*M or 500 *μ*M for 24 h. Specimens were evaluated using FITC-Annexin V (Biotium Inc.), which can identify apoptotic cells by their green fluorescence by binding to phosphatidylserine (PS) under conditions where PS is translocated from the inner (cytoplasmic) leaflet of the plasma membrane to the outer (cell surface) leaflet soon after the induction of apoptosis. In brief, live specimens were washed twice with 1× binding buffer and then incubated for 30 min with 5% Annexin V in 1× binding buffer. After Annexin V labeling, the specimens were fixed in 10% formalin in 0.1 M PBS for 2 h. After being rinsed with 0.1 M PBS, SGNs and auditory nerve fibers were routinely labeled with Alexa Fluor 555-conjugated neurofilament 200 to identify the neurons, while the cochlear HCs were routinely stained with 0.5% Texas Red-X phalloidin (Invitrogen T7471) in PBS for 1 h to separately label the stereocilia and cuticular plate of the HCs. Samples were washed three times in PBS and mounted in glycerin on glass slides.

Specimens were examined under a confocal microscope (Zeiss LSM-510 meta, step size 0.5 *μ*m per slice) with appropriate filters to detect the red fluorescence of Alexa Fluor 555 (absorption 555 nm, emission 565 nm) or Texas Red-X (excitation 591 nm, emission 608 nm), to detect the green fluorescence of Alexa 488 or FITC (excitation 495 nm, emission 519 nm).

For sensory HCs quantifications, cochlear cultures whose stereocilia and cuticular plate had been fluorescently labeled were observed under a fluorescent microscope (Zeiss, Axioskop) equipped with appropriate filters. The number of missing cochlear HCs for each 0.24 mm segment was counted over the entire length of the organ of Corti from apex to base, as described previously [36, 41, 42]. A cochleogram was constructed to determine the percentage of inner hair cells (IHCs) and outer hair cells (OHCs) as a function of the percentage of the distance from the apex to the base. Using custom cochleogram software and laboratory norms from control rats, we plotted the average (*n* = 5/condition) percentage of HCs missing as a function of the percentage of the distance from the apex of the cochlea for each experimental group. Specimens were also photographed with a confocal microscope (Bio-Rad MRC1024). Images were processed with Zeiss LSM Image Examiner (Carl Zeiss MicroImaging GmbH) and Adobe Photoshop (version 5.5), as described previously [35, 37, 39–44].

To quantify the fragmented SGNs, the size of the SGNs in each experimental condition was examined with a Confocal LSM Image Examiner as described previously [42]. In brief, multiple layers of confocal images at a magnification of ×630 were collected.

Multiple layers of the images were selected and merged into a single layer such that the SGNs with the largest cross sectional area were measured. To avoid repeated measurements, the images were collected from different regions along the entire length of Rosenthal's canal in the modiolus, and there was an interval of 20 *μ*m between each merged layer. A polygon was drawn around the perimeter of the cell body of all distinguishable SGNs, and the Zeiss LSM Image Examiner (version: 4,0,0,91) automatically calculated the enclosed area. Intact SGNs and degenerating SGNs with associated fragmentation were distinguished and counted respectively in all experimental groups (150 SGNs per group). All data were evaluated for statistical significance with SigmaStat (version 3.5.0.54).

The same quantification for the positive FITC-Annexin V labeling of SGNs was performed. Confocal images from three cochleae that had been treated with 50 *μ*M ouabain and three cochleae treated that had been treated with 500 *μ*M ouabain were observed layer by layer (50 SGNs per cochlea, and a total of 150 SGNs per condition). The positive and negative FITC-Annexin V labeled SGNs were counted respectively. Data of FITC-Annexin V positive SGNs were analyzed by one-way ANOVA followed by Newman-Keuls post hoc analyses (GraphPad Prism 5 software).

### 2.4. Quantitative RT-PCR Apoptosis-Focused Gene Arrays

According to the results of our preliminary experiments, ouabain-induced apoptotic signals were present only in the HCs, and did not appear in cochlear supporting cells at the early stage. To detect apoptosis-related genes in cochlear HCs by PCR array, it was challenging to collect pure HCs since they were most usually associated with cochlear supporting cells. Considering that HCs only occupy a very small proportion of the total cell population on the cochlear basilar membrane, any changes in gene expression in HCs might be concealed by those unchanged supporting cells present in the whole cochlear basilar membrane when testing for gene expression changes by PCR array. Therefore, the apoptosis-related genes in HCs were not explored by PCR array in the current study. By contrast, most cells in Rosenthal's canal are SGNs, which almost entirely expressed apoptotic signals through Annexin V detection at the early stage after ouabain treatment. Therefore, all of the SGNs in the entire Rosenthal's canal were carefully separated along the inner edge of the basilar membrane for the Rat Apoptosis RT^2^ Profiler PCR Array (SABiosciences, PARN-012A) detection in the current study. The numbers of cultures for each condition were as follows: 4 h, control (20), 2 h, 500 *μ*M ouabain (20), and 4 h, 500 *μ*M ouabain (20).

The isolated SGNs in Rosenthal's canal were cultured with/without ouabain to determine the expression of apoptosis-related genes by qRT-PCR array that was designed in 96 wells for the detection of 84 key apoptosis-related genes. Sample preparation and determination of the RT-PCR array were similar to those described in our previous studies [42, 45]. In brief, after culture with/without ouabain, SGNs were homogenized in buffer RLT. Total RNA was isolated and extracted using an RNA extraction kit (RNeasy Lipid Tissue Mini Kit, QIAGEN 74804) according to the manufacturer's protocols. Each sample of purified tRNA was diluted 1 : 100 in RNase-free water and examined on a spectrophotometer to test the purity and concentration of RNA used for synthesis of first-strand complementary DNA (cDNA; RT^2^ First Strand Kit, SABiosciences, C-03). The concentration of tRNA was used to ensure a consistent quantity of tRNA (0.5 *μ*g in this experiment), which was used for cDNA synthesis across all experimental conditions.

Expression of 84 apoptosis-related genes was evaluated in control and ouabain-treated (500 *μ*M ouabain, 2 or 4 h duration) SGNs samples in a 96-well plate (including five housekeeping and seven control genes) by the Rat Apoptosis RT^2^ Profiler PCR Array (SABiosciences, PARN-012A). Apoptosis gene arrays were processed according to the manufacturer's instructions. RT^2^ Real-TimeTM SYBR Green/fluorescein PCR Master Mix (included in the kit) was used to monitor the fluorescent signal during each cycle of the PCR reaction. The apoptosis arrays were evaluated on a MyiQ TM Single-Color Real-Time PCR Detection System (BIO-RAD, Model No. MyiQ TM Optical Module). Each PCR reaction started with an initial denaturation cycle at 95°C for 10 min, followed by 40 cycles consisting of 15 s at 95°C for denaturation and 1 min at 60°C for annealing. Each experimental condition was repeated three times. The threshold for calculating cycle threshold (Ct) values was calculated automatically using MyiQ software (version: 1.0.410). When comparing multiple tests, a fixed threshold was assigned manually, as suggested by the manufacturer. The relative expression of each of the 84 apoptosis-related genes was calculated using the ΔΔCt method [46].

Ct values were transferred into Microsoft Excel (Microsoft Office 2003) and analyzed with Web-Based PCR Array Data Analysis tools: (http://www.sabiosciences.com/pcr/arrayanalysis.php, SABiosciences) to determine the fold change and *P* value of each gene. Those changes (*P* < 0.05, ≥2-fold change) were defined as biologically relevant changes [42].

## 3. Results

### 3.1. Degeneration in the Cochlear HCs and Auditory Nerve Fibers

**Figure 1:**
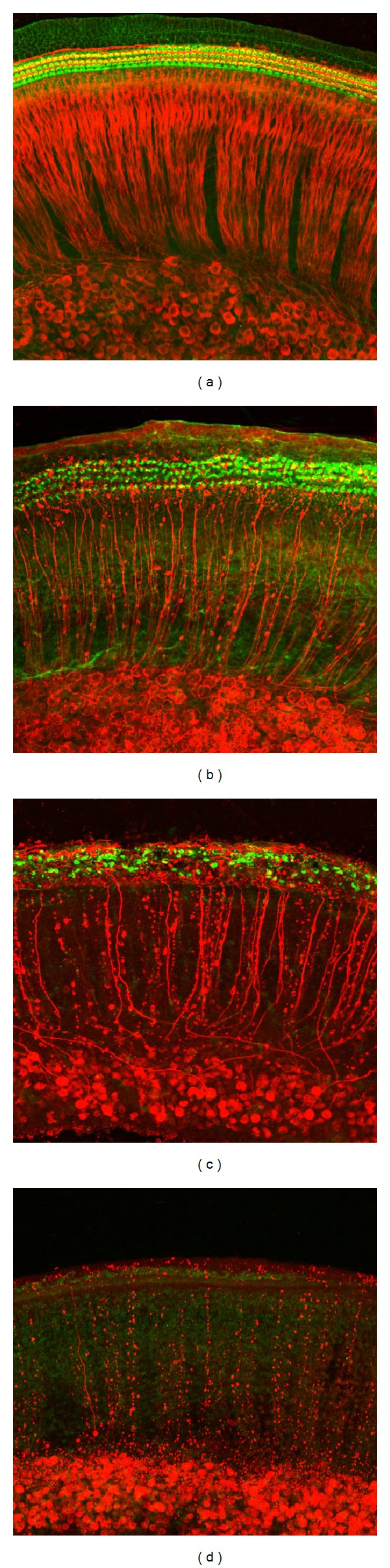
Organotypic cultures from the middle cochleae 72 h after ouabain treatment. The HCs were stained with Alexa Fluor 488-phalloidin. Auditory nerve fibers and SGNs were labeled with an antibody directed against neurofilament 200 and an Alexa Fluor 555 conjugated secondary antibody. (a) Cochlear organotypic culture from postnatal day 3 rat that was cultured for 72 h under normal conditions. (b) Rat cochlear cultures treated with 50 *μ*M ouabain for 72 h. Note that all HCs were intact, whereas the density of the auditory nerve fibers was greatly reduced as compared with the normal control in (a). (c) Cochlear explants were treated with 500 *μ*M ouabain for 72 h. Many SGNs and nerve fibers degenerated and significant percentage's of HCs were missing. (d) Cochlear cultures treated with 1 mM ouabain for 72 h resulted in complete destruction of both SGNs and HCs in the cochlea (Fluorescence microscope ×200).

The photomicrograph (Figure 1(a)) showed the orderly arrangement of cochlear outer hair cells and inner hair cells with green fluorescent labeling and auditory nerve fibers with red fluorescent labeling. In addition, SGNs with red fluorescence in a normal cochlear explant cultured for 72 h are shown. This data indicated that the sensory HCs and auditory innervations had been regularly growing for at least three days in the cochlear organotypic primary culture system. Cochlear HCs were intact (Figure 1(b)), whereas the density of auditory nerve fibers was greatly reduced 72 h after 50 *μ*M ouabain treatment. Adding 500 *μ*M of ouabain to the culture medium for 72 h resulted in severe degeneration in SGNs and auditory nerve fibers with massive loss of cochlear HCs (Figure 1(c)). Increasing the ouabain dose to 1000 *μ*M destroyed all of the SGNs, auditory nerve fibers, and cochlear HCs (Figure 1(d)).

### 3.2. Ouabain Dose-Response Effects and Cochlear HCs

**Figure 2:**
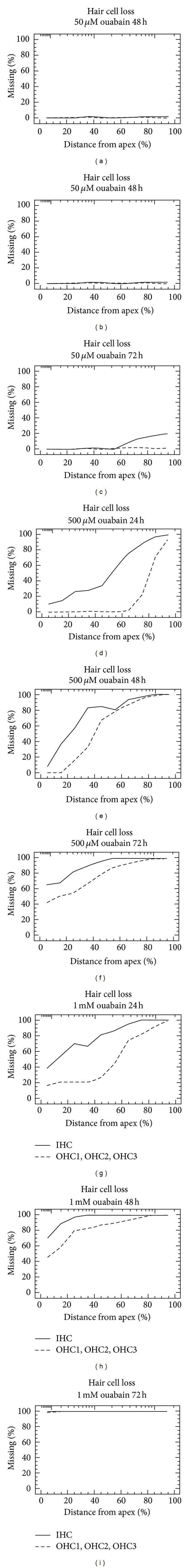
Representative cochleograms that show the percentage of missing IHCs and OHCs following ouabain treatment. No evidence of missing HCs was found ((a) and (b)). Panels (a) and (b) show the mean cochleograms (average of *N* = 5) at 24 h and 48 h following 50 *μ*M ouabain treatment, respectively. However, approximately 20% of IHCs were missing at the basal turn of the cochlea (c), which shows the mean cochleogram at 72 h after treatment with 50 *μ*M ouabain. (d), (e), and (f) show the mean cochleograms at 24 h, 48 h, and 72 h after treatment with 500 *μ*M ouabain, respectively. Note that the loss in HCs progresses from the base to the apex in a time-dependent manner. In addition, loss in IHCs was greater than OHCs. (g), (h), and (i) represent the mean cochleograms at 24 h, 48 h, and 72 h after treatment with 1000 *μ*M ouabain. At 24 h after 1000 *μ*M ouabain treatment, approximately 85% of IHCs and 50% of OHCs were missing (g). When the culture period was extended to 48 h, most IHCs and OHCs were no longer present, with the notable exception of the tip of the apex (h). Treatment with 1000 *μ*M ouabain for 72 h destroyed 100% of HCs (i).

The cochlear HCs were treated with 50 *μ*M ouabain for 24 h and 48 h presented as normal appearance (Figures 2(a) and 2(b)). However, 72 h after 50 *μ*M ouabain treatment, about 20% of the IHCs were missing in the basal turn, but all of the OHCs were found to be intact (Figure 2(c)). Treatment with 500 *μ*M ouabain for 24 h destroyed more than 50% of the IHCs and 20% of the OHCs. The loss of HCs typically began at the basal turn and migrated towards the apical turn of the cochlea (Figure 2(d)). When the duration of the culture was extended to 48 h, a dose of 500 *μ*M ouabain destroyed 70% of the IHCs and 50% of the OHCs (Figure 2(e)). Extending the duration of the culture to 72 h, treatment with 500 *μ*M ouabain destroyed 90% of the IHCs and 80% of the OHCs (Figure 2(f)). Increasing the concentration of ouabain to 1000 *μ*M resulted in the destruction of more than 80% of the IHCs and about 50% of the OHCs 24 h after ouabain treatment (Figure 2(g)). By contrast, 90%–100% of cochlear HCs were missing 48 h and 72 h after 1000 *μ*M ouabain treatment, respectively (Figures 2(h) and 2(i)). The graphic panels of cochleograms (Figure 2) clearly showed that ouabain-induced destruction of cochlear HCs expanded from the basal turn to the apical turn, and that destruction of IHCs was more severe and occurred earlier than that seen with OHCs.

### 3.3. Damage in the Soma of SGNs

The profile and the density of SGNs were all within the normal range in normal control cochleae from 24–72 h in standard culture conditions. By contrast, extensive damage in SGNs was found at 48 h and 72 h after ouabain treatment, even with a low dose of ouabain treatment at 50 *μ*M.

**Figure 3:**
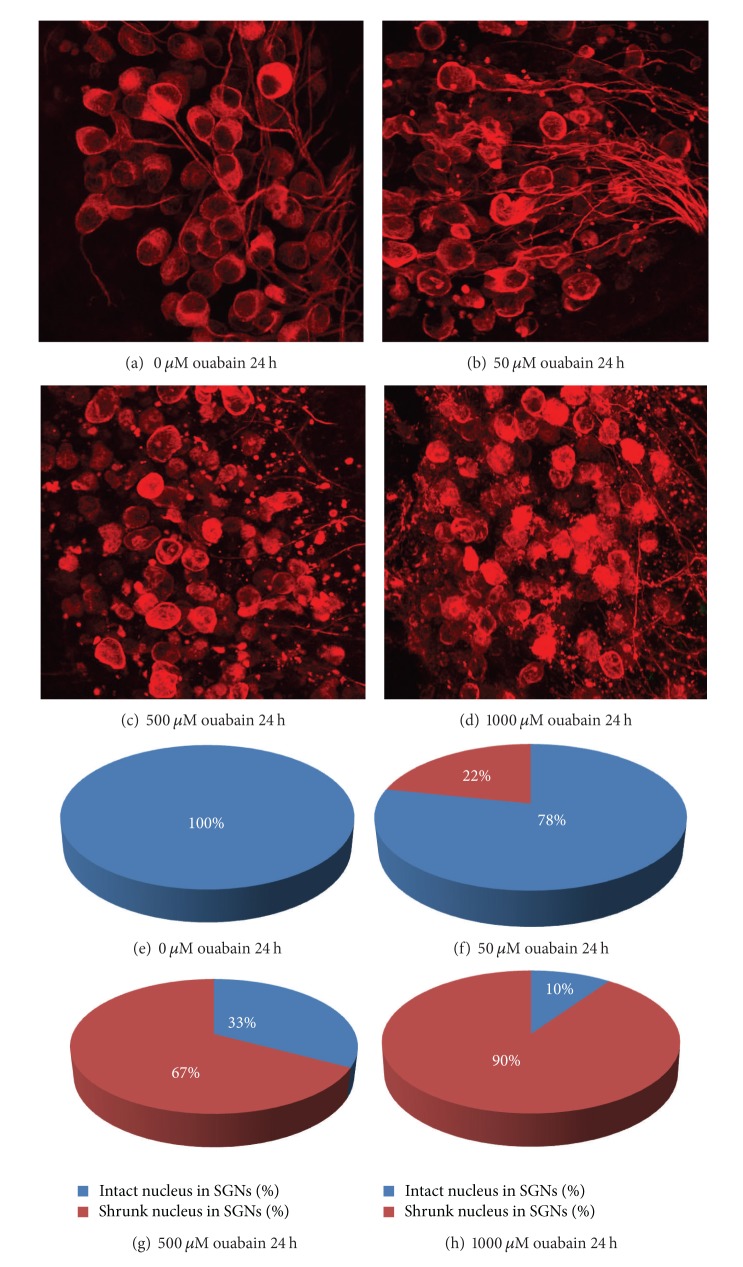
Photomicrographs show SGNs at 24 h following ouabain treatment (0–1000 *μ*M). (a) SGNs were cultured for 24 h in a standard culture medium without ouabain. Note that SGNs and auditory nerve fibers were intact. (b) 50 *μ*M ouabain treatment for 24 h caused condensation of the body in some SGNs. (c) 500 *μ*M ouabain for 24 h resulted in soma condensation or fragmentation in most SGNs. (d) All SGNs and auditory nerve fibers were destroyed 24 h after 1000 *μ*M ouabain treatment (fluorescence microscope magnification ×600). Specimens were labeled with an antibody directed against neurofilament 200 and Alexa Fluor 555 conjugated secondary antibody (red). (e) At 24 h after culture with standard medium without ouabain, all SGNs were intact. (f) At 24 h after 500 *μ*M ouabain, 22% SGNs were condensed or shrunk. (g) At 24 h after 500 *μ*M ouabain, the percentage of degenerating SGNs with cell shrinkage was greatly increased with 67% showing degeneration. (h) At 24 h after 1000 *μ*M ouabain, 90% SGNs were present with morphological apoptotic features seen and characteristic cell condensation, cell shrinkage, and/or nuclear/cellular fragmentation.

To adequately express the neurotoxic response in SGNs, four images were chosen from 24 h cultures with/without ouabain treatment (Figure 3). We found a normal soma of SGNs and intact auditory nerve fibers that were cultured in a standard culture medium without ouabain for 24 h (Figure 3(a)). By contrast, Figure 3(b) showed that treatment with 50 uM ouabain for 24 h induced pathological changes that included cell condensation or fragmentation in many SGNs. When the concentrations of ouabain increased to 500 *μ*M or 1000 *μ*M for 24 h in the cultures, most SGNs were completely destroyed (Figures 3(c) and 3(d)).

After 50 *μ*M ouabain treatment for 24, 48, and 72 h, neuron size measurements showed that the number of condensed SGNs was increased to 22% (Figure 3(f)), 43%, and 86%, respectively. Treatment with 500 *μ*M ouabain for 24, 48, and 72 h increased the number of condensed SGNs to 67% (Figure 3(g)), 91%, and 100%, respectively. When the concentration of ouabain increased to 1000 *μ*M, 90% of the SGNs were found to be condensed or fragmented 24 h after culture (Figure 3(h)), while nearly 100% of the SGNs were fragmented at 48 h and 72 h after treatment with 1000 *μ*M ouabain, respectively. Treatment with different doses of ouabain for various periods caused a statistically significant increase in SGN fragmentation relative to the controls (as determined by One way ANOVA, Tukey post-hoc test, *P* < 0.01).

### 3.4. Apoptotic Signals in Cochlear HCs and SGNs

**Figure 4:**
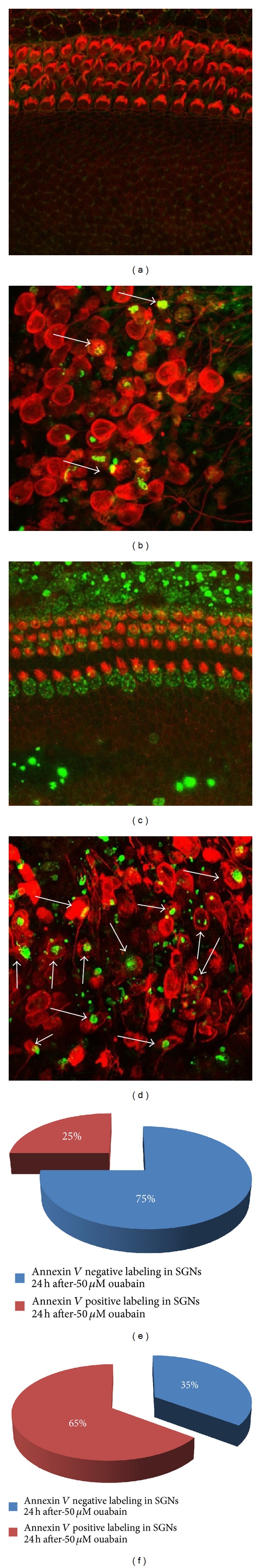
Apoptotic labeling in HCs and SGNs after ouabain treatment in cochlear organotypic cultures. (a) There was no evidence of positive Annexin V labeling in HCs 24 h after 50 *μ*M ouabain treatment, although the stereocilia were disarrayed on the HCs. (b) In the same culture conditions as (a), apoptotic signals were detected in some SGNs (arrow). (c) Annexin V positive labeling was seen in HCs and the supporting cells in the region of the lateral sulcus 24 h after 500 *μ*M ouabain treatment. (d) In the same culture conditions as (c), with 500 *μ*M ouabain, most SGNs presented with positive Annexin V expression (arrows) showing apoptotic features (fluorescence microscope at a magnification of ×600). (e) At 24 h after 50 *μ*M ouabain treatment, a quarter of the SGNs exhibited FITC-Annexin V positive labeling. (f) At 24 h after 500 *μ*M ouabain treatment, the FITC-Annexin V positive labeling of SGNs was increased to 65%.

**Figure 5:**
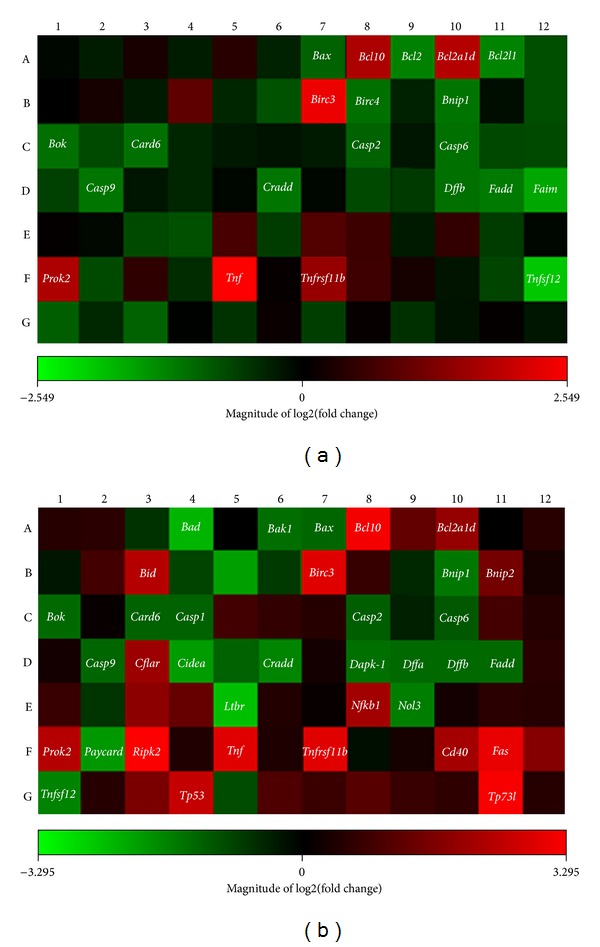
Visualization of rat apoptosis RT^2^ ProfilerTM PCR array following 500 *μ*M ouabain treatment. (a) 20 apoptosis-related genes were upregulated (red) or downregulated (green) 2 h after 500 *μ*M ouabain treatment. (b) 35 apoptosis-related genes were upregulated (red) or downregulated (green) at 4 h after 500 *μ*M ouabain treatment. The number of significantly altered apoptosis-related genes rapidly increased within 2 h.

Considering that cell shrinkage and condensation were detected by regular staining with neurofilament-200 immunolabeling in ouabain-treated SGNs, we used FITC-Annexin V techniques for the detection of apoptosis in cochlear HCs and SGNs. At 24 h after 50 *μ*M ouabain treatment, the FITC-Annexin V labeling was negative in the cochlear HCs, despite disarrayed stereocilia on the HCs that were detected at this time (Figure 4(a)). However, the positive apoptotic signals (green fluorescence) were found in the condensed or fragmented SGNs (Figure 4(b)) 24 h after 50 *μ*M ouabain treatment. At 24 h after 500 *μ*M ouabain treatment, despite all HCs being present in the cochlea apical turn (Figure 2(d)), the F ITC-Annexin V labeled membranes had significantly increased in most HCs in the apical region and in some supporting cells in the outer sulcus region (Figure 4(c)). Most SGNs had greatly shrunk and showed obvious positive expression of Annexin V 24 h after 500 *μ*M ouabain treatment (Figure 4(d)). By quantitative observations, 35 positive FITC-Annexin V labeled SGNs were detected from 140 SGNs 24 h after 50 *μ*M ouaba in treatment, and the positive labeling was equivalent to 25% observed SGNs (Figure 4(e)). It is worthwhile to note that when the concentration of ouabain increased to 500 *μ*M, 89 FITC-Annexin V positively labeled SGNs were found in 137 SGNs 24 h after ouabain treatment, an observation that equaled the 65% observed SGNs (see Figure 5F). By statistical analysis, a significant increase in the number of positively stained FITC-Annexin V labeled SGNs was detected 24 h after 50 *μ*M or 500 *μ*M ouabain treatment (One-way ANOVA, *F* = 301.6, *P* < 0.0001, and Newman-Keuls post hoc analysis, *P* < 0.05) as compared with normal controls. There was also a significant difference between the 50 *μ*M ouabain treated group and the 500 *μ*M ouabain treated group (Newman-Keuls post hoc analysis, *P* < 0.05). The Annexin V positive response in cochlear HCs and SGNs suggested that ouabain-induced cell destruction was involved in cell apoptosis. In addition, the apoptotic signals appeared in the SGNs earlier than in the cochlear HCs.

### 3.5. Changes in the Expression of Apoptosis-Related Genes after Ouabain Treatment

Using Rat Apoptosis RT^2^ ProfilerTM PCR Array, SGNs treated with 500 *μ*M ouabain for 2 h or 4 h were assayed. At 2 h after ouabain treatment (Figure 5(a)), a significant increase or decrease in gene expression was found in 20 apoptosis-related genes (*P* < 0.05), while the remaining 64 genes were unaltered (*P* > 0.05). However, in the samples treated with 500 *μ*M ouabain for 4 h (Figure 5(b)), expression in 35 genes was markedly changed (*P* < 0.05), while the remaining 49 genes were unchanged (*P* > 0.05). The significant changes in gene expression are listed in Tables 1 and 2, respectively. The new response genes found after 4 h of treatment with 500 *μ*M ouabain suggested that more apoptosis-related genes were activated after 2 h of 500 *μ*M ouabain treatment.

At 2 h after 500 *μ*M ouabain treatment, expression in 6 genes (*Bcl10*, *Bcl2a1d, Birc3, Prok2, Tnf*, and *Tnfrsf11b*) increased, but the expression in a further 14 genes (*Bcl2, Bcl2l1, Birc4, Bnip1, Bok, Card6, Casp2, Casp6, Casp9, Cradd, Dffb, Fadd, Faim, *and *Tnfsf12*) had decreased. In those altered 20 genes, 10 were anti-apoptotic genes or negative regulator genes (*Bcl10, Bcl2a1d, Birc3, Prok2, Tnf, Bcl2, Bcl2l1, Birc4, Bnip1,* and *Faim*), while the other 10 genes were pro-apoptotic or positive regulator genes (*Tnfrsf11b, Bok, Card 6, Casp2, Casp6, Casp9, Cradd, Dffb, Fadd,* and *Tnfsf12*).

The 20 genes described above could be classified into the TNF-ligand or TNF-receptor family (*Tnf, Tnfsf12, Tnfrsf11b, Prok2,* and *Faim*), Bcl-2 family (*Bcl10, Bcl2, Bcl2a1d, Bcl2l1, Bnip1, Bok*), the Caspase family (*Casp2, Casp6,* and *Casp9*), the IAP family (*Birc4* and *Birc3*), the CARD family (*Bcl10, Birc3, Birc4, Card6, Casp2, Casp9,* and *Cradd*), the death-domain or death-effector-domain family (*Cradd, Fadd, Prok2,* and *Tnfrsf11b*), CIDE-domain family (*Dffb*), and the p53- and DNA-damage-induced apoptosis family of genes (*Bcl2, Bcl2a1d, Bcl2l1, Casp1, Casp2, Casp6, Casp9, Cradd, Fadd, Faim, Prok2,* and *Tnf*), respectively. To summarize the apoptotic pathways in the early-altered 20 genes 2 h after ouabain treatment, we found that 25% of the genes were directly involved in the TNF-ligand or TNF-receptor family, 30% belonged to the Bcl-2 family, 15% were members of Caspase family, 10% were in the IAP family, 35% belonged to CARD family, 20% were part of the death-domain or death-effector-domain family, 5% were in the CIDE-domain family, and 60% were related to p53- and DNA-damage-induced apoptosis family of genes. The total percentage was over 100% because many genes were involved in multiple apoptotic pathways. For example, genes in the *Tnf *family were also important apoptotic regulators in the p53-signaling pathway. *Casp6 *was another example; it is a member of the Caspase family but also plays an important role in the CARD and the p53 families. Therefore, one altered gene may affect multiple apoptotic pathways.

At 4 h after 500 *μ*M ouabain treatment, expression in 15 genes was greatly upregulated (*P* < 0.05); these genes were *Bcl10, Bcl2a1d, Bid, Birc3, Bnip2, Cflar, Nfkb1, Prok2, Ripk2, Tnf, Tnfrsf11b, Tnfrsf5 (CD40), Tnfrsf6 (Fas), Tp53*, and *Tp73l*. Another 20 genes were significantly downregulated (*Bad, Bak1, Bax, Bnip1, Bok, Card6, Casp1, Casp2, Casp6, Casp9, Cidea, Cradd, Dapk-1, Dffa, Dffb, Fadd, Ltbr, Nol3, Pycard, and Tnfsf12*) (*P* < 0.05).

In those altered 35 genes, 17 genes were anti-apoptotic or negative regulator genes (*Bcl10, Bcl2a1d, Birc3, Bnip1, Bnip2, Cflar, Cidea, Dapk-1, Nfkb1, Nol3, Prok2, Ripk2, Tnf, CD40, Fas, Tp53,* and *Tp73l*), and another 18 were pro-apoptotic or positive regulators (*Bid, Tnfrsf11b, Bad, Bak1, Bax, Bok, Card6, Casp1, Casp2, Casp6, Casp9, Cradd, Dffa, Dffb, Fadd, Ltbr, Pycard, *and *Tnfsf12*).

These 35 genes can be classified into the TNF-ligand or TNF-receptor family (*Ltbr, Prok2, CD40, Fas, Dapk*-1*, Tnf, Tnfsf12,* and *Tnfrsf11b*), the Bcl-2 family (*Bad, Bak1, Bax, Bcl10, Bcl2a1d, Bid, Bnip1, Bnip2,* and *Bok)*, the Caspases and its regulators family (*Bax*, *Cflar, Casp1, Casp2, Casp6, Casp9, Pycard,* and *Tp53*), the IAP family (*Birc3*), CARD family (*Bcl10, Birc3, Card6, Casp1, Casp2, Casp9, Cradd, Nol3, Pycard,* and *Ripk2*), death-domain or death-effector domain-family (*Cflar, Cradd, Dapk1, Fadd, Nfkb1, Pycard, Tnfrsf11b, CD40,* and *Fas*), CIDE-domain family (*Cidea, Dffa,* and *Dffb*), and p53 and DNA-damage-induced apoptosis family (*Bad, Bax, Bid, Casp6, Casp9, Cradd, Fadd, Nfkb1, Prok2, Pycard, Tnf, Tp53,* and *Tp731*), respectively.

In the collected data from the above 35 altered apoptosis-related genes following 4 h treatment with ouabain, 20% of the genes were in the TNF-ligand or TNF-receptor family, 26% of the genes belonged to the Bcl-2 family, 16% were members of the Caspase family, 3% were in the IAP family, 26% belonged to the CARD family, 29% were part of the death-domain or death-effector-domain family, 6% were in the CIDE-domain family, and 43% were related to the p53- and DNA-damage-induced apoptosis family of genes. As described previously, the total percentage involving various apoptotic pathways exceeded 100% due to many genes being involved in multiple apoptotic pathways.

**Figure 6:**
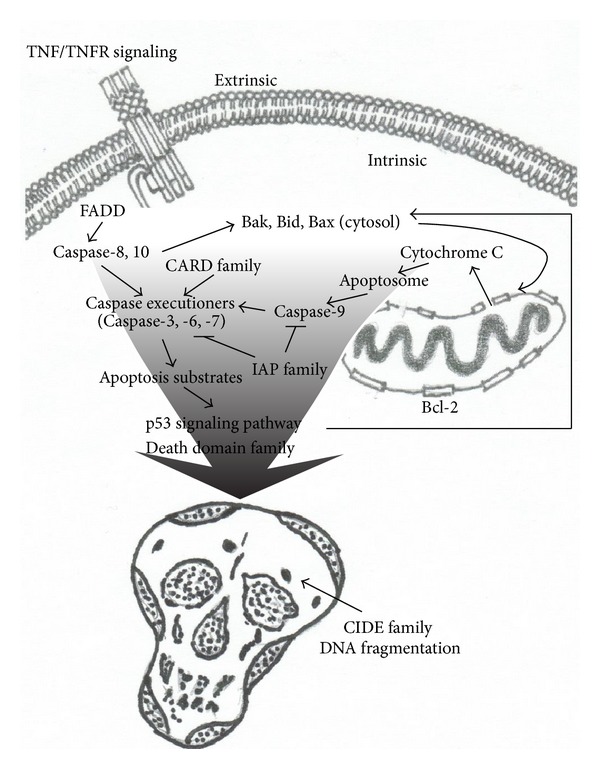
Major apoptotic pathways in SGNs induced by oubain treatment. Genes that respond to intrinsic stimulation are believed to be involved in the mitochondrial pathway, and two major factors appear to be important, namely cytochrome C and apoptosis inducing factor (AIF). The pathway began with Caspase-9 activation, which was followed by executioner Caspases that resulted in proteolytic activity and nuclear damage (DNA fragmentation). The extrinsic pathway-responding genes were triggered first by cell-surface occupation of receptors belonging to the TNF/TNF R superfamily, which activate Fas causing the recruitment of Fas-associated protein with death domain (FADD). Then, Caspase-8 cleaves other pro-Caspases, which effectively initiates a Caspase cascade that ultimately drives apoptosis. Caspase-8 activation can also activate Bak, Bid, and Bax, which enhance mitochondrial damage and promote leakage of cytochrome C. Since many groups of genes are involved in the intrinsic and extrinsic pathways, many of which belong to the p53 superfamily, it was inevitable that the p53 signaling pathway was involved in ouabain-induced apoptosis.

The major pathways induced by 500 *μ*M ouabain treatment were displayed in a cartoon illustration (see Figure 6). This suggested that ouabain-induced apoptotic signaling might have initiated both extrinsic and intrinsic stimulated pathways and finally converged to the p53-signaling pathway.

## 4. Discussion

Ouabain has served as a common tool in many previous investigations to explore the physiological and pathological changes in the cochlea in adult mammals *in vivo *[4, 27, 29, 30, 33]. However, ototoxic effects of ouabain *in vivo *vary according to the different kinds of mammalian species under study. In order to explain these different ototoxic effects of ouabain *in vivo*, further studies were warranted.

The observations from the current study provide the first evidence of the ototoxic effects of ouabain *in vitro*. Our microscopic observations revealed a sequence of ouabain-induced pathology in the cochlea that started with prior degeneration in SGNs and auditory nerve fibers 72 h following lowdose (50 *μ*M) ouabain treatment, while most cochlear HCs were intact. This may have indicated that the toxic effects of ouabain occurred earlier in peripheral auditory neurons than in the cochlear sensory HCs. However, when the concentration of ouabain was increased to 500 *μ*M or 1000 *μ*M, most cochlear HCs were also destroyed. This suggested that ouabain was also very toxic to cochlear HCs *in vitro *when its concentration was extremely high.

Thus, our findings suggested that SGNs in postnatal day 3, rats were more vulnerable to ouabain than the cochlear HCs in culture conditions. To pursue the probable causes, it should be noted that the Na^+^/K^+^-ATPase *α* and *β* subunit isoforms (*α* subunit *α*1, *α*3, and *β* subunit *β*1, *β*2) were strongly expressed in SGNs in newborn rats but were absent in cochlear HCs after birth. The expression of Na^+^/K^+^-ATPase *α* and *β* subunit isoforms initially appeared in the cochlear HCs and usually does so seven days after birth and reaches normal levels around postnatal day 21 [14, 17]. Moreover, the Na^+^/K^+^-ATPase *α*3 subunit was most sensitive to ouabain that was strongly expressed only in SGNs, especially during the period immediately after birth in the rat [47]. Therefore, this might represent one possible approach to induce the susceptibility of SGNs in response to injury by ouabain at postnatal day 3 in cochlear organotypic cultures.

Ouabain-induced degeneration of cochlear HCs occurs in a dose- and time-dependent manner, exhibiting a stereotypic pattern of damage that begins at the base of the cochlea and progresses toward the apex. The destruction of gradient HCs was consistent with other ototoxic reagents, such as that provided by gentamicin. Gentamicin generally damaged the cochlear OHCs [48]. However, ouabain preferentially targets the cochlear IHCs in culture conditions. This kind of damage pattern was similar to the ototoxic effects of dimethyl sulphoxide and manganese, in which damage to the IHCs was more prominent than OHCs lesions *in vitro *[41].

Considering that IHCs only connect with type I SGNs, whereas type I SGNs are the major target of ouabain in gerbil, mouse, and rat [27–29, 33], the susceptible and missing IHCs suggests that ouabain selectively affects the type I auditory nervous system in the cochlea including type I SGNs and their innervated IHCs. Based on the first finding in the current study, we can only suggest the involvement of this potential phenomenon, and we can only suggest further explanations for the specific mechanisms in the present paper.

Prior studies have shown that an extremely low dose of ouabain may protect SGNs from apoptosis when a concentration of ouabain as low as 10 nM is applied [49, 50]. In the current study, the lowest dose of ouabain that we used was 50 *μ*M, which was 5000 times higher than 10 nM. This concentration of ouabain clearly led to apoptosis in SGNs. The Annexin V positive labeling in SGNs and cochlear HCs revealed that ouabain-induced destruction in both SGNs and cochlear HCs were specifically related to or characterized by apoptosis. These findings are consistent with previous findings that ouabain induces apoptosis in SGNs *in vivo *[27, 29]. When the cochlear explants were treated with ouabain at a concentration of 500 *μ*M for 24 h, many HCs at the apex remained viable yet displayed intense Annexin V labeling. This indicated that the surviving HCs were already in the initial phases or the process of programmed cell death and doomed to perish soon after by apoptosis.

To investigate the apoptotic mechanisms involved in ouabain-induced cellular apoptosis, a quantitative RT-PCR apoptosis-focused gene array (84 apoptosis-related genes) was used to assess changes in apoptosis-related gene expression in SGNs treated with 500 *μ*M ouabain. For gene array testing in the present study, the cochlear explants were harvested at 2 h or 4 h after ouabain treatment and before cell destruction. The purpose for the two designed schedules was conside red so that attempts could be made to determine the earliest responding genes, which may have been closely related to the initial apoptotic response.

In present study, we only detected gene expression by apoptosis-focused PCR array in SGNs but did not conduct the same test in cochlear HCs. The reason lies in the fact that most cells in Rosenthal's canal are SGNs at the early stage without too much interaction from other cell types. In addition, many apoptotic signals are early expressed in most SGNs, so that it is relatively uncomplicated to detect changes in apoptosis-related genes from the relatively pure neurons. By contrast, there are various cell types on cochlear basilar membrane, and the percentages of cochlear HCs are less than 10% of all cell types on cochlear basilar membrane. Moreover, apoptotic signals were preferentially expressed in cochlear HCs after ouabain treatment, whereas apoptotic signals did not appear in most supporting cells. To avoid the unresponsive background from most supporting cells, the gene array was not applied to the tissue of cochlear basilar membrane due to its inconsistent response from miscellaneous cell types.

At 2 h after ouabain treatment, 20 apoptosis-related genes were significantly changed and were either upregulated or downregulated, while the remaining 64 genes remained unaltered. By contrast, the number of altered genes increased to 35 at 4 h after 500 *μ*M ouabain treatment. This might indicate that the rapid changes in gene expression were in response to the release of apoptotic signals from SGNs by ouabain injury. In addition, the rapid increase of responding genes from 2 h to 4 h after ouabain treatment might suggest that a greater interaction was triggered, which provoked a chain reaction from more apoptosis-related genes following ouabain treatment. For example, 2 h after 500 *μ*M ouabain treatment, only one proapoptotic gene, *Bok*, which is a member of the Bcl-2 family, was altered. By contrast, 4 h after 500 *μ*M ouabain treatment, the number of pro-apoptotic genes in the Bcl-2 family increased to five. Whatever the up-regulation or down-regulation events, changes in gene expression can always be considered as evidence of the cell responding to apoptotic stimuli.

No matter how different the quantity of altered genes between 2 h and 4 h after ouabain exposure is, the altered genes in SGNs were involved in the following apoptotic families: TNF-ligand or TNF-receptor family, Bcl-2 family, Caspase family, IAP family, CARD family, death-domain or death-effector-domain family, CIDE-domain family, and p53- and DNA-damage-induced apoptosis. Despite the overlap in gene expression between 2 h and 4 h after ouabain, the new altered genes in specimens 4 h after ouabain treatment can be considered as apoptotic response. However, the altered genes at 2 h after ouabain were the earliest responding genes that might approximate the actual factors in the initial mechanisms of the onset of apoptosis.

We synthesized the relevant data to determine the major apoptotic pathways in ouabain-induced apoptosis, especially in those apoptotic pathways with a high percentage of altered genes. Based on our observations, 25% of the altered genes were members of the TNF family 2 h after 500 *μ*M ouabain treatment. Considering that the tumor necrosis factor (TNF) ligands or receptors are located on the cell membrane, the gene changes in the TNF family are believed to be related to membranous damage and to extrinsic attack or intrinsic injury [49, 51–53]. The TNF family can also trigger multiple signaling pathways, such as activation of a Caspase cascade that might indirectly shift the balance in the Bcl2 family and activate the p53 family [49, 54, 55].

In those altered genes 2 h after 500 *μ*M ouabain exposure, 30% were members of the Bcl-2 family. Since Bcl-2 is a family that governs mitochondrial outer membrane permeabilization, changes in the Bcl-2 family are usually considered in relation to mitochondrial damage. *Caspase-9-*mediated killing is initiated only through mitochondrial damage, which leads to the release of cytochrome C into the cytosol [11, 36, 43, 56–62]. We found significant changes in gene expression of *Casp9*. The decreased gene expression in *Casp9 *may reflect a tentative response from *Casp9 *by ouabain, but upregulated antiapoptotic genes, such as *Birc3* in the IAP family, might also suppress it, and *Birc2* is an antiapoptotic gene that specifically blocks cytochrome C release [62]. Further, many genes in the Bcl-2 family are also crucial members of the p53 signaling pathway. Therefore, as a prognostic indicator of p53 activity, Bcl-2 is thought to interact with the p53 family [63–66].

Furthermore, 35% of the total altered genes 2 h after 500 *μ*M ouabain treatment were CARD members. The full name of the CARD family is Caspase recruitment domain family. As the name suggests, the altered genes in the CARD family are related to the Caspase family and have a partial bearing on the p53 family.

Moreover, 60% of the total changed genes 2 h after ouabain were involved in the p53 pathway, which reached the maximum percentage of total altered genes. This result was consistent with previous studies that showed that the p53 pathway was part of an important process of apoptotic execution in ouabain-induced apoptosis [66–68]. In addition, many members of the Bcl-2 family, TNF family, CARD family, Caspase family, IAP family, and death-domain family are also important regulators for the governance of the p53 family [54, 63, 69, 70]. Considering that the p53 pathway and its complex can be stimulated by a wide variety of signals in response to cellular stress, the genes in the p53 family are considered to execute ouabain-induced apoptosis in SGNs.

In summary, ouabain-induced damage to SGNs and cochlear HCs occurred in a dose- and time-dependent manner, whereas the damage in SGNs occurred earlier and more severe than that in cochlear HCs. A feature of cochlear HC lesions induced by ouabain treatment was a gradient progression from base to apex. Interestingly, the IHCs were more susceptible to ouabain toxicity than OHCs, which was characterized by cochleograms. The pattern of ouabain-induced cell death in both SGNs and the HCs was apoptosis and was confirmed by Annexin V labeling. The data of quantitative RT-PCR apoptosis-focused gene array showed that multiple pathways and some of the altered genes were pro-apoptotic, and some were antiapoptotic. However, irrespective of their effect, the changes in those responding genes generally suggested that the cells were responding to the stimuli of specific apoptotic signals. In accordance with the large percentage of responding genes at an early stage, TNF, Bcl-2, death domain, and p53 families, TNF, and Bcl-2 were believed to occur in response to extrinsic stimuli from membranous damage and intrinsic stimuli from mitochondrial damage, which initiated the death receptor-mediated signaling pathway and apoptotic mitochondrial pathway, respectively. However, the apoptotic signaling release from both extrinsic and intrinsic attacks then evoked a wide network of response signaling and in multiple genes. This broad array of response signaling triggered the p53-dependent pathway to execute and complete cellular apoptosis. 

## Figures and Tables

**Table 1:** Altered gene name, description, functional grouping, and fold change, with *P* value, 2 h after 500 *µ*M ouabain treatment.

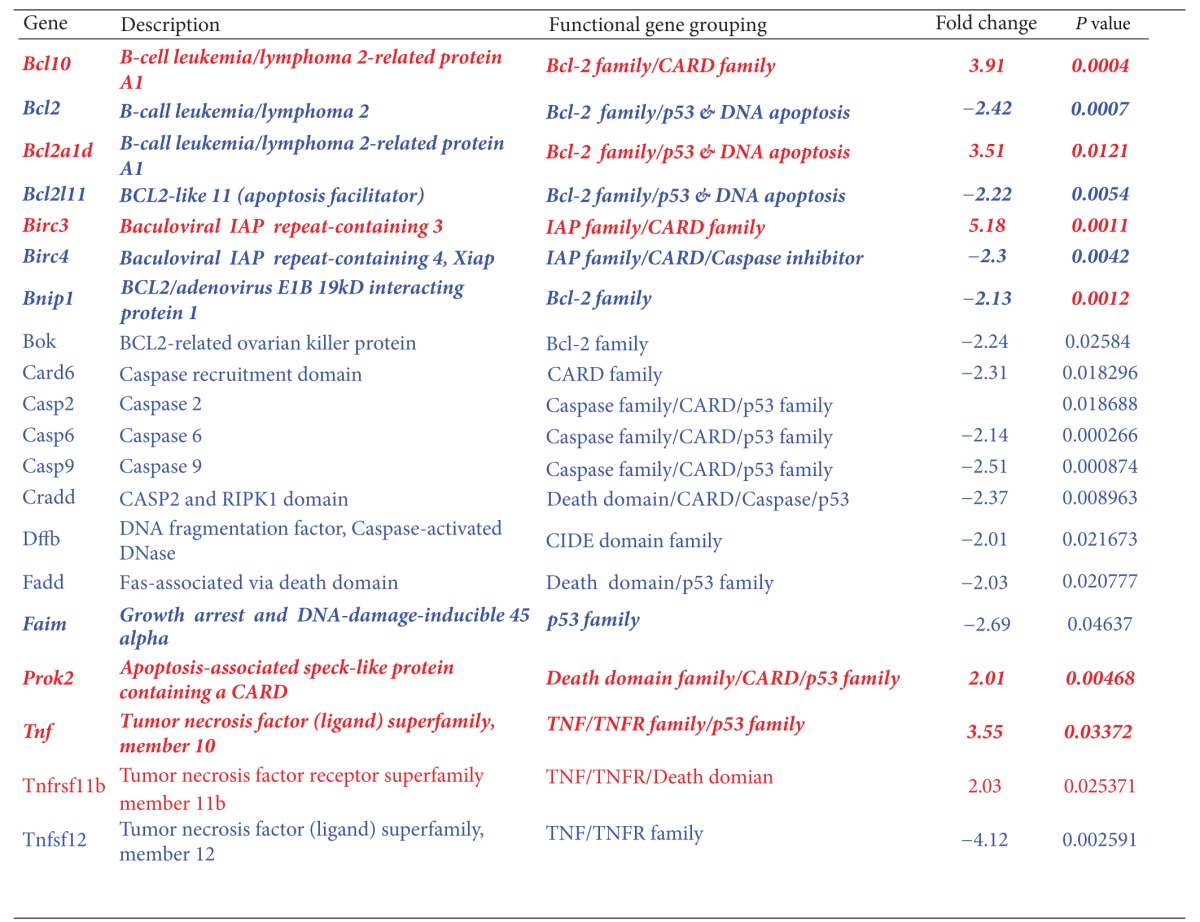

Antiapoptotic genes were marked with italics in boldface, while proapoptotic genes were written in regular letters. Genes with red text were overexpressed, whereas genes with blue text were down-regulation.

**Table 2:** Altered gene name, description, functional grouping, and fold change, with *P* value, 4 h after 500 *µ*M ouabain treatment.

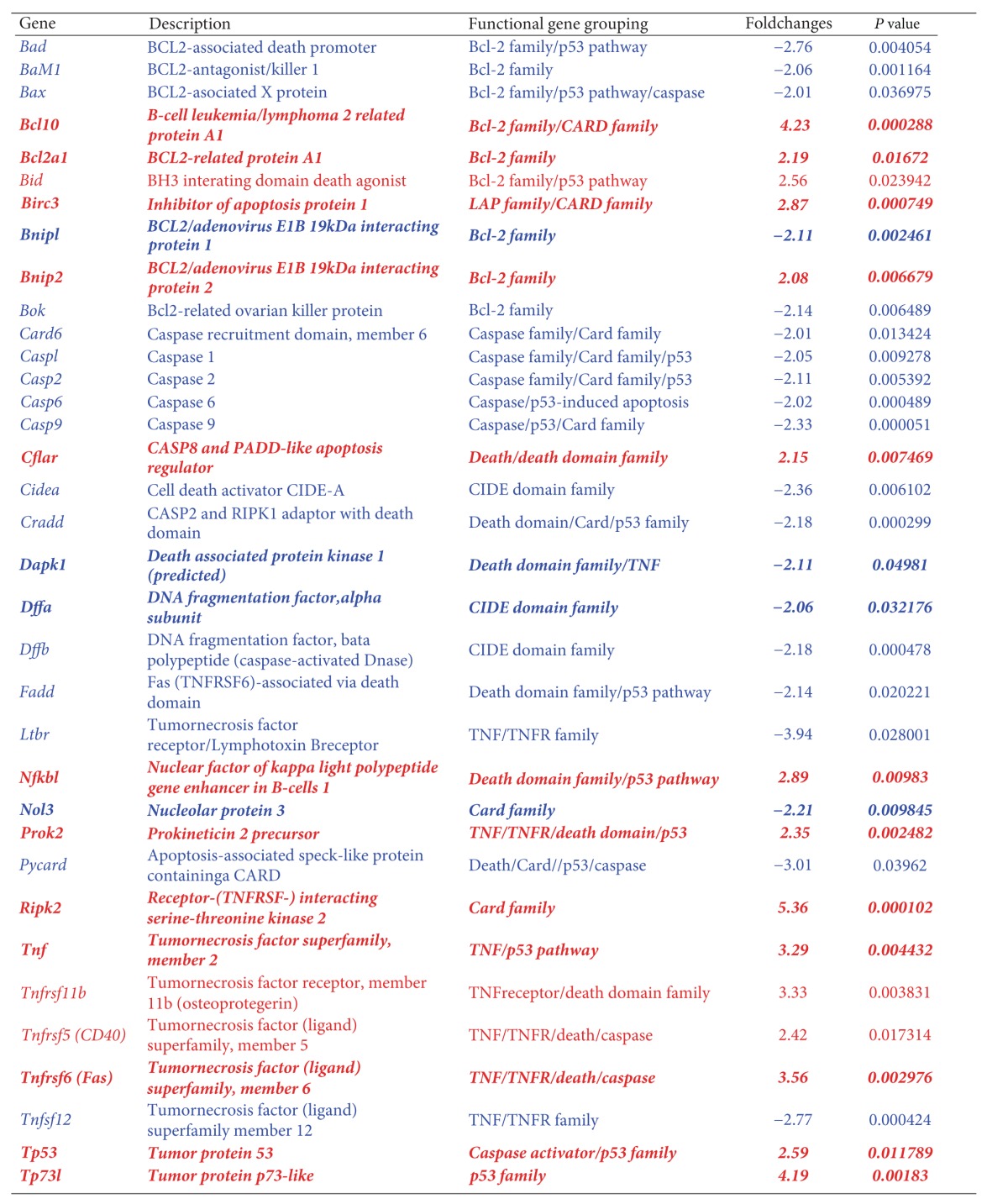

Antiapoptotic genes were marked with italics in boldface, while proapoptotic genes were written in regular letters. Genes with red text were overexpressed, whereas genes with blue text were downregulation.

## References

[1] Rajasekaran AK, Rajasekaran SA (2003). Role of Na-K-ATPase in the assembly of tight junctions. *The American Journal of Physiology—Renal Physiology*.

[2] Kaplan JH (2002). Biochemistry of Na,K-ATPase. *Annual Review of Biochemistry*.

[3] Geering K, Beggah A, Good P (1996). Oligomerization and maturation of Na,K-ATPase: functional interaction of the cytoplasmic NH2 terminus of the *β* subunit with the *α* subunit. *Journal of Cell Biology*.

[4] Hamada M, Kimura RS (1999). Morphological changes induced by administration of a Na^+^,K^+^-ATPase inhibitor in normal and hydropic inner ears of the guinea pig. *Acta Oto-Laryngologica*.

[5] Ichimiya I, Adams JC, Kimura RS (1994). Immunolocalization of Na^+^,K^+^-ATPase, Ca^++^-ATPase, calcium-binding proteins, and carbonic anhydrase in the guinea pig inner ear. *Acta Oto-Laryngologica*.

[6] Kuijpers W, Bonting SL (1969). Studies on (Na^+^-K^+^)-activated ATPase XXIV. Localization and properties of ATPase in the inner ear of the guinea pig. *Biochimica et Biophysica Acta*.

[7] Cate WJFT, Curtis LM, Rarey KE (1994). Na,K-ATPase *α* and *β* subunit isoform distribution in the rat cochlear and vestibular tissuese. *Hearing Research*.

[8] Nakazawa K, Spicer SS, Schulte BA (1995). Ultrastructural localization of Na,K-ATPase in the gerbil cochlea. *Journal of Histochemistry and Cytochemistry*.

[9] McLean WJ, Smith KA, Glowatzki E, Pyott SJ (2009). Distribution of the Na,K-ATPase *α* subunit in the rat spiral ganglion and organ of corti. *Journal of the Association for Research in Otolaryngology*.

[10] Pitovski DZ, Kerr TP (2002). Sodium- and potassium-activated ATPase in the mammalian vestibular system. *Hearing Research*.

[11] Schulte BA, Adams JC (1989). Distribution of immunoreactive Na^+^,K^+^-ATPase in gerbil cochlea. *Journal of Histochemistry and Cytochemistry*.

[12] Souter M, Forge A (1998). Intercellular junctional maturation in the stria vascularis: possible association with onset and rise of endocochlear potential. *Hearing Research*.

[13] Okamura H, Spicer SS, Schulte BA (2001). Developmental expression of monocarboxylate transporter in the gerbil inner ear. *Neuroscience*.

[14] Peters TA, Kuijpers W, Curfs JHAJ (2001). Occurrence of NaK-ATPase isoforms during rat inner ear development and functional implications. *European Archives of Oto-Rhino-Laryngology*.

[15] Xia A, Kikuchi T, Hozawa K, Katori Y, Takasaka T (1999). Expression of connexin 26 and Na,K-ATPase in the developing mouse cochlear lateral wall: functional implications. *Brain Research*.

[16] Erichsen S, Berger S, Schmid W, Stierna P, Hultcrantz M (2001). Na,K-ATPase expression in the mouse cochlea is not dependent on the mineralocorticoid receptor. *Hearing Research*.

[17] Zuo J, Curtis LM, Yao X, Cate WJFT, Rarey KE (1995). Expression of Na, K-ATPase *α* and *β* isoforms in the neonatal rat cochlea. *Acta Oto-Laryngologica*.

[18] Sagara T, Furukawa H, Makishima K, Fujimoto S (1995). Differentiation of the rat stria vascularis. *Hearing Research*.

[19] Kuijpers W, van der Vleuten AC, Bontino SL (1967). Cochlear function and sodium and potassium activated adenosine triphosphatase. *Science*.

[20] Konishi T, Mendelsohn M (1970). Effect of ouabain on cochlear potentials and endolymph composition in guinea pigs. *Acta Oto-Laryngologica*.

[21] Kuijpers W, Bonting SL (1970). The cochlear potentials—I: the effect of ouabain on the cochlear potentials of the guinea pig. *Pflügers Archiv*.

[22] Sellick PM, Johnstone BM (1974). Differential effects of ouabain and ethacrynic acid on the labyrinthine potentials. *Pflügers Archiv*.

[23] Bosher SK (1980). The effects of inhibition of the strial Na^+^-K^+^-activated ATPase by perilymphatic ouabain in the guinea pig. *Acta Oto-Laryngologica*.

[24] Marcus DC, Marcus NY, Thalmann R (1981). Changes in cation contents of stria vascularis with ouabain and potassium-free perfusion. *Hearing Research*.

[25] Wangemann P, Liu J, Marcus DC (1995). Ion transport mechanisms responsible for K^+^ secretion and the transepithelial voltage across marginal cells of stria vascularis in vitro. *Hearing Research*.

[26] Konishi T, Salt AN (1980). Permeability to potassium of the endolymph-perilymph barrier and its possible relation to hair cell function. *Experimental Brain Research*.

[27] Lang H, Schulte BA, Schmiedt RA (2005). Ouabain induces apoptotic cell death in type I spiral ganglion neurons, but not type II neurons. *Journal of the Association for Research in Otolaryngology*.

[28] Lang H, Li M, Kilpatrick LA (2011). Sox2 up-regulation and glial cell proliferation following degeneration of spiral ganglion neurons in the adult mouse inner ear. *Journal of the Association for Research in Otolaryngology*.

[29] Schmiedt RA, Okamura HO, Lang H, Schulte BA (2002). Ouabain application to the round window of the gerbil cochlea: a model of auditory neuropathy and apoptosis. *Journal of the Association for Research in Otolaryngology*.

[30] Wang LE, Cao KL, Yin SK, Wang Z, Chen Z (2006). Cochlear function after selective spiral ganglion cells degeneration induced by ouabain. *Chinese Medical Journal*.

[31] Rivolta MN, Li H, Heller S (2006). Generation of inner ear cell types from embryonic stem cells. *Methods in Molecular Biology*.

[32] Matsuoka AJ, Kondo T, Miyamoto RT, Hashino E (2007). Enhanced survival of bone-marrow-derived pluripotent stem cells in an animal model of auditory neuropathy. *Laryngoscope*.

[33] Lang H, Schulte BA, Goddard JC (2008). Transplantation of mouse embryonic stem cells into the cochlea of an auditory-neuropathy animal model: effects of timing after injury. *Journal of the Association for Research in Otolaryngology*.

[34] F u Y, Ding D, Jiang H, Salvi R (2012). Ouabain-induced cochlear degeneration in rat. *Neurotoxicity Research*.

[35] Ding D, Stracher A, Salvi RJ (2002). Leupeptin protects cochlear and vestibular hair cells from gentamicin ototoxicity. *Hearing Research*.

[36] Ding D, He J, Allman BL (2011). Cisplatin ototoxicity in rat cochlear organotypic cultures. *Hearing Research*.

[37] McFadden SL, Ding D, Salvemini D, Salvi RJ (2003). M40403, a superoxide dismutase mimetic, protects cochlear hair cells from gentamicin, but not cisplatin toxicity. *Toxicology and Applied Pharmacology*.

[38] Zhang M, Liu W, Ding D, Salvi R (2003). Pifithrin-*α* supresses p53 and protects cochlear and vestibular hair cells from cisplatin-induced apoptosis. *Neuroscience*.

[39] Corbacella E, Lanzoni I, Ding D, Previati M, Salvi R (2004). Minocycline attenuates gentamicin induced hair cell loss in neonatal cochlear cultures. *Hearing Research*.

[40] Nicotera TM, Ding D, McFadden SL, Salvemini D, Salvi R (2004). Paraquat-induced hair cell damage and protection with the superoxide dismutase mimetic M40403. *Audiology and Neuro-Otology*.

[41] Qi W, Ding D, Salvi RJ (2008). Cytotoxic effects of dimethyl sulphoxide (DMSO) on cochlear organotypic cultures. *Hearing Research*.

[42] Wei L, Ding D, Salvi R (2010). Salicylate-induced degeneration of cochlea spiral ganglion neurons-apoptosis signaling. *Neuroscience*.

[43] Ding D, Jiang H, Wang P, Salvi R (2007). Cell death after co-administration of cisplatin and ethacrynic acid. *Hearing research*.

[44] Ding D, Jiang H, Salvi RJ (2010). Mechanisms of rapid sensory hair-cell death following co-administration of gentamicin and ethacrynic acid. *Hearing Research*.

[45] Hu BH, Cai Q, Manohar S (2009). Differential expression of apoptosis-related genes in the cochlea of noise-exposed rats. *Neuroscience*.

[46] Livak KJ, Schmittgen TD (2001). Analysis of relative gene expression data using real-time quantitative PCR and the 2-ΔΔCT method. *Methods*.

[47] Hara Y, Urayama O, Kawakami K (1988). The third type of alpha-subunit of Na,K-ATPase. *Progress in clinical and biological research*.

[48] Fetoni AR, Eramo SL, Rolesi R, Troiani D, Paludetti G (2012). Antioxidant treatment with coenzyme Q-ter in prevention of gentamycin ototoxicity in an animal model. *Acta Otorhinolaryngologica Italica*.

[49] Golden WC, Martin LJ (2006). Low-dose ouabain protects against excitotoxic apoptosis and up-regulates nuclear Bcl-2 in vivo. *Neuroscience*.

[50] Wei Y, Xiao H, Jiang Y, Yang C, Zheng N (2009). Low dose of ouabain protects injury of spiral ganglion neurons in vitro. *Lin Chung Er Bi Yan Hou Tou Jing Wai Ke Za Zhi*.

[51] Segura MF, Sole C, Pascual M (2007). The long form of Fas apoptotic inhibitory molecule is expressed specifically in neurons and protects them against death receptor-triggered apoptosis. *Journal of Neuroscience*.

[52] Humphreys EH, Williams KT, Adams DH, Afford SC (2010). Primary and malignant cholangiocytes undergo CD40 mediated fas dependent apoptosis, but are insensitive to direct activation with exogenous fas ligand. *PLoS ONE*.

[53] Tigno-Aranjuez JT, Asara JM, Abbott DW (2010). Inhibition of RIP2’s tyrosine kinase activity limits NOD2-driven cytokine responses. *Genes and Development*.

[54] Haupt S, Berger M, Goldberg Z, Haupt Y (2003). Apoptosis—the p53 network. *Journal of Cell Science*.

[55] Persons DL, Yazlovitskaya EM, Pelling JC (2000). Effect of extracellular signal-regulated kinase on p53 accumulation in response to cisplatin. *The Journal of Biological Chemistry*.

[56] Krajewski S, Krajewska M, Ellerby LM (1999). Release of caspase-9 from mitochondria during neuronal apoptosis and cerebral ischemia. *Proceedings of the National Academy of Sciences of the United States of America*.

[57] Harrison LRE, Micha D, Brandenburg M (2011). Hypoxic human cancer cells are sensitized to BH-3 mimetic-induced apoptosis via downregulation of the Bcl-2 protein Mcl-1. *Journal of Clinical Investigation*.

[58] Du H, Wolf J, Schafer B, Moldoveanu T, Chipuk JE, Kuwana T (2011). BH3 domains other than bim and bid can directly activate bax/bak. *The Journal of Biological Chemistry*.

[59] Carter BZ, Qiu YH, Zhang N (2011). Expression of ARC (apoptosis repressor with caspase recruitment domain), an antiapoptotic protein, is strongly prognostic in AML. *Blood*.

[60] Bandyopadhyay S, Chiang CY, Srivastava J (2010). A human MAP kinase interactome. *Nature Methods*.

[61] Brunelle JK, Letai A (2009). Control of mitochondrial apoptosis by the Bcl-2 family. *Journal of Cell Science*.

[62] Deveraux QL, Roy N, Stennicke HR (1998). IAPs block apoptotic events induced by caspase-8 and cytochrome c by direct inhibition of distinct caspases. *EMBO Journal*.

[63] Park SA, Park HJ, Lee BI, Ahn YH, Kim SU, Choi KS (2001). Bcl-2 blocks cisplatin-induced apoptosis by suppression of ERK-mediated p53 accumulation in B104 cells. *Molecular Brain Research*.

[64] Palacios G, Moll UM (2006). Mitochondrially targeted wild-type p53 suppresses growth of mutant p53 lymphomas in vivo. *Oncogene*.

[65] Jang TH, Bae JY, Park OK (2010). Identification and analysis of dominant negative mutants of RAIDD and PIDD. *Biochimica et Biophysica Acta*.

[66] Kulikov A, Eva A, Kirch U, Boldyrev A, Scheiner-Bobis G (2007). Ouabain activates signaling pathways associated with cell death in human neuroblastoma. *Biochimica Et Biophysica Acta*.

[67] Pshezhetsky AV (2007). Proteomic analysis of vascular smooth muscle cells treated with ouabain. *Methods in Molecular Biology*.

[68] Taurin S, Seyrantepe V, Orlov SN (2002). Proteome analysis and functional expression identify mortalin as an antiapoptotic gene induced by elevation of [Na^+^]i/[K^+^]i ratio in cultured vascular smooth muscle cells. *Circulation Research*.

[69] Park HH, Wu H (2006). Crystal structure of RAIDD death domain implicates potential mechanism of PIDDosome assembly. *Journal of Molecular Biology*.

[70] Tong QS, Zheng LD, Wang L (2005). Downregulation of XIAP expression induces apoptosis and enhances chemotherapeutic sensitivity in human gastric cancer cells. *Cancer Gene Therapy*.

